# Crystal structure of (N^C) cyclo­metalated Au^III^ diazide at 100 K

**DOI:** 10.1107/S2056989020012955

**Published:** 2020-10-09

**Authors:** Volodymyr Levchenko, Sigurd Øien-Ødegaard, David Wragg, Mats Tilset

**Affiliations:** a Department of Chemistry and Center for Materials Science and Nanotechnology (SMN), University of Oslo, PO Box 1126 Blindern, N-0318 Oslo, Norway

**Keywords:** crystal structure, azide, gold(III), cyclo­metallated, *trans* influence

## Abstract

The title compound, an (N^C)-cyclo­metalated gold(III) diazide, namely, di­azido­[5-eth­oxy­carbonyl-2-(5-eth­oxy­carbonyl­pyridin-2-yl)phenyl-κ^2^ *C* ^1^,*N*]gold(III), [Au(C_17_H_16_NO_4_)(N_3_)_2_] or Au(ppy^Et^)(N_3_)_2_, was synthesized by reacting Au(ppy^Et^)Cl_2_ with NaN_3_ in water for 24 h. The complex has been structurally characterized and features a gold center with a square-planar environment. The Au—N(azide) bond lengths are significantly different depending on the influence of the atom *trans* to the azide group [Au—N(*trans* to C) of 2.067 (2) Å *versus* Au—N(*trans* to N) of 2.042 (2) Å]. The azide groups are twisted in-and-out of plane by 56.2 (2)°.

## Abstract 2

The title compound, an (N^C)-cyclo­metalated gold(III) diazide, namely, di­azido­[5-eth­oxy­carbonyl-2-(5-eth­oxy­carbonyl­pyridin-2-yl)phenyl-κ^2^ *C* ^1^,*N*]gold(III), [Au(C_17_H_16_NO_4_)(N_3_)_2_] or Au(ppy^Et^)(N_3_)_2_, was synthesized by reacting Au(ppy^Et^)Cl_2_ with NaN_3_ in water for 24 h. The complex features a gold center with a square-planar environment.

## Chemical context   

**Figure 1:**
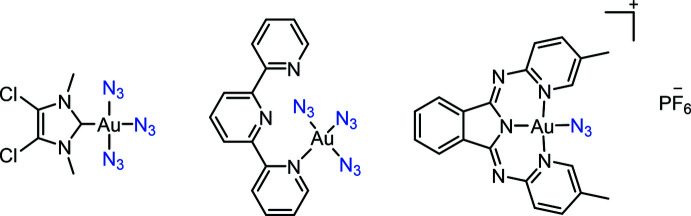
Au^III^–azide complexes reported in the literature

Among gold azide complexes, Au^I^ have dominated over Au^III^ azides (Del Castillo *et al.*, 2011 ▸; Powers *et al.*, 2015 ▸; Partyka *et al.*, 2007 ▸). Until now, only three examples of Au^III^ azide complexes have been reported (Fig. 1 ▸). The reported compounds feature the N-heterocyclic carbene complex and pyridine coordinated Au–triazide groups (Schuh *et al.*, 2016 ▸; Peng *et al.*, 2019 ▸) as well as cationic cyclo­metalated monoazide (Roth *et al.*, 2016 ▸). To the best of our knowledge, a cyclo­metalated phenyl pyridine Au^III^ azide complex has not been reported before.
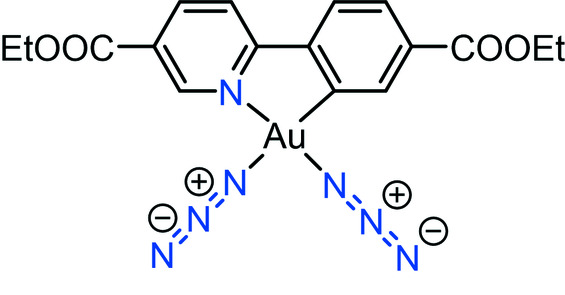


## Structural commentary   

**Figure 2:**
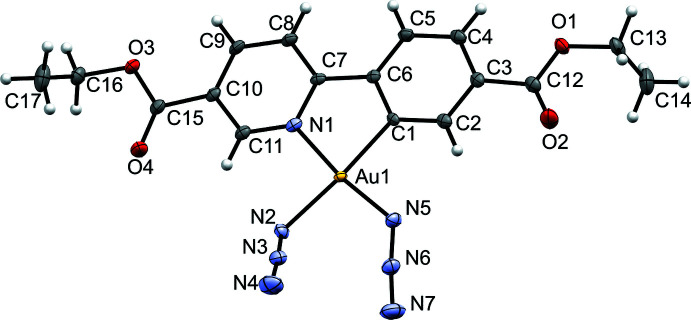
Mol­ecular structure of the title compound. Displacement ellipsoids are drawn at the 50% probability level.

**Figure 3:**
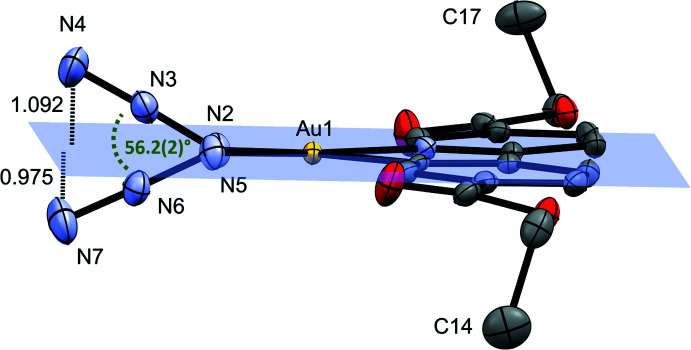
Mutual orientation of the azide groups with respect to the metalacycle plane.

The mol­ecular structure of Au(ppy^Et^)(N_3_)_2_ (**2**) is shown in Fig. 2 ▸. The complex forms monoclinic crystals belonging to the space group *P*2_1_/*c* and crystallizes with one mol­ecule in the asymmetric unit. The solid-state structure of **2** displays a square-planar coordination geometry, as expected for the *d* ^8^ electron configuration of the Au^III^ center. The Au—N bond length *trans* to the pyridine N atom [2.042 (2) Å] is shorter than the one *trans* to the C atom [2.067 (2) Å], indicating the stronger *trans* influence of the phenyl carbon atom. N—N bond distances in the azide ligands are in line with reported literature values (Dori *et al.*, 1973 ▸) with shorter terminal N—N bond lengths compared to the inter­nal ones (1.150 *vs* 1.200 Å, on average). The N—N—N angles [174.7 (3) and 173.8 (3)°] deviate only slightly from the expected linear arrangement and the Au—N—N angles of 118.7 (2)° and 119.2 (2)° for the azide groups *trans* to N and C, respectively, indicate the expected bent coordination of these ligands. The azide groups are twisted by 56.2 (2)° with respect to each other, and point in-and-out of the plane with distances of 1.092 (2) Å for the terminal N atom *trans* to C and 0.975 (2) Å for the terminal N atom *trans* to the pyridine N atom (Fig. 3 ▸). The pyridine and benzene rings are essentially coplanar, the angle formed by their mean planes being 3.64 (10)°.

## Supra­molecular features   

**Figure 4:**
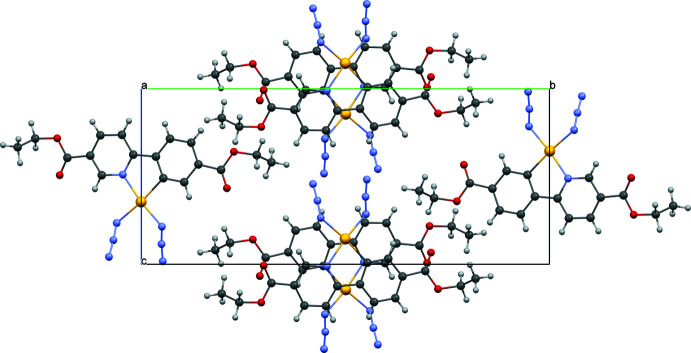
Crystal packing of the title compound viewed along the *a* axis.

The title crystal structure features infinite stacking chains along the [100] direction. The neighboring mol­ecules within the stack are related by inversion. The mean plane of the core of the complex mol­ecule including the Au atom, both aromatic rings and two N atoms of azide groups attached to the Au atom form an angle with the *a*-axis direction of 69.53 (2)°. The distances between these planes of neighboring mol­ecules within the stack are 3.331 (1) and 3.314 (1) Å (Fig. 4 ▸).

## Database survey   

A search was performed in the Cambridge Structural Database (CSD version 5.41; Groom *et al.*, 2016 ▸) with the following constraints: an Au^III^ complex featuring a phenyl­pyridine backbone and two additional non-cyclic ligands bonding to Au through N or C. Fourteen structures were found to match this motif. The features of the title structure resemble those observed in the structures found in this database survey, *e.g*. an observable *trans* effect (distance Au—*L trans* to N is always shorter than that *trans* to C), Au—C bond lengths are shorter than the Au—N ones and angles around the Au^III^ center are close to 90°.

## Synthesis and crystallization   

**Figure 5:**
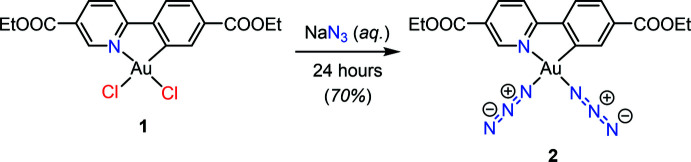
Synthesis of the title compound.

The reaction scheme for the synthesis of the title compound is provided in Fig. 5 ▸. The gold complex Au(ppy^Et^)Cl_2_ (**1**) was prepared according to previously published procedure (Levchenko *et al.*, 2020 ▸). Complex **1** (70 mg, 0.124 mmol, 1 equiv.) was stirred with sodium azide (64.5 mg, 1 mmol, 8 equiv.) in water for 24 h at room temperature. The solids were recovered by filtration, washed with large excess of water and dried in air giving 50 mg (70%) of **2** as a white solid. Needle-like crystals were obtained by slow diffusion of cyclo­hexane into a solution of the product in CH_2_Cl_2_ containing few drops of acetone. **^1^H NMR** (600 MHz, DMSO-*d*_6_): δ_H_ 9.28 (*s*, 1H), 8.82 (*d*, *J* = 8.4 Hz, 1H), 8.61 (*d*, *J* = 8.4 Hz, 1H), 8.24 (*d*, *J* = 8.2 Hz, 1H), 8.07–8.02 (*m*, 2H), 4.45 (*dd*, *J* = 12.5, 5.4 Hz, 2H), 4.38 (*q*, *J* = 7.0 Hz, 2H), 1.37 (*dt*, *J* = 13.8, 7.2 Hz, 6H). **^13^C NMR** (151 MHz, DMSO-*d*_6_): δ_C_ 165.1, 164.5, 162.3, 148.9, 147.0, 146.3, 143.8, 132.5, 129.6, 128.2, 127.4, 127.1, 122.8, 62.3, 61.5, 14.1, 14.0. **MS** (ESI, CH_3_OH): *m*/*z* = 591.091 ([*M* − N_3_ + OCH_3_ + Na]^+^, 100), 602.082 ([*M* + Na]^+^, 9). **HRMS** (CH_3_OH): calculated for C_18_H_19_AuN_4_O_5_Na^+^ [*M* − N_3_ + OCH_3_ + Na]^+^ 591.0913, found 591.0914 (Δ 0.00 ppm). Calculated for C_17_H_16_AuN_7_O_4_Na^+^ [*M* + Na]^+^ 602.0822, found 602.0821 (Δ 0.10 ppm).

## Refinement   

Crystal data, data collection and structure refinement details are summarized in Table 1 ▸. *OLEX2* was used as user inter­face (Dolomanov *et al.*, 2009 ▸). All hydrogen atoms were placed in calculated positions with C—H = 0.95-0.99 Å and refined as riding with fixed isotropic displacement parameters [*U*_iso_(H) = 1.2-1.5*U*_eq_(C)].

## Supplementary Material

Crystal structure: contains datablock(s) I. DOI: 10.1107/S2056989020012955/yk2139sup1.cif

Structure factors: contains datablock(s) I. DOI: 10.1107/S2056989020012955/yk2139Isup2.hkl

CCDC reference: 2033308

Additional supporting information:  crystallographic information; 3D view; checkCIF report

## Figures and Tables

**Table 1:** Experimental details

Crystal data	
Chemical formula	[Au(C_17_H_16_NO_4_)(N_3_)_2_]
*M*_r_	579.33
Crystal system, space group	Monoclinic, *P*2_1_/*c*
Temperature (K)	100
*a*, *b*, *c* (Å)	7.0788 (5), 24.6279 (16), 10.5840 (7)
β (°)	91.059 (1)
*V* (Å^3^)	1844.9 (2)
*Z*	4
Radiation type	Mo *K*α
μ (mm^−1^)	8.02
Crystal size (mm)	0.2 × 0.03 × 0.01

Data collection	
Diffractometer	Bruker D8 Photon 100 area detector
Absorption correction	Multi-scan (*SADABS*; Bruker, 2018 ▸)
*T*_min_, *T*_max_	0.554, 0.746
No. of measured, independent and observed [*I* > 2σ(*I*)] reflections	37671, 5655, 4677
*R*_int_	0.023
(sin θ/λ)_max_ (Å^−1^)	0.715

Refinement	
*R*[*F* ^2^ > 2σ(*F* ^2^)], *wR*(*F* ^2^), *S*	0.018, 0.036, 1.09
No. of reflections	5655
No. of parameters	264
H-atom treatment	H-atom parameters constrained
Δρ_max_, Δρ_min_ (e Å^−3^)	0.92, −1.10

Computer programs: *APEX3* (Bruker, 2018 ▸), SHELXT (Sheldrick, 2015*a* ▸), *SHELXL* (Sheldrick, 2015*b* ▸), *Mercury* (Macrae *et al.*, 2020 ▸), *enCIFer* (Allen *et al.*, 2004 ▸).

## supplementary crystallographic information

### Crystal data

**Table:**

[Au(C_17_H_16_NO_4_)(N_3_)_2_]	*F*(000) = 1112
*M**_r_* = 579.33	*D*_x_ = 2.086 Mg m^−^^3^
Monoclinic, *P*2_1_/*c*	Mo *K*α radiation, λ = 0.71073 Å
*a* = 7.0788 (5) Å	Cell parameters from 9519 reflections
*b* = 24.6279 (16) Å	θ = 2.5–30.6°
*c* = 10.5840 (7) Å	µ = 8.02 mm^−^^1^
β = 91.059 (1)°	*T* = 100 K
*V* = 1844.9 (2) Å^3^	Needle, colorless
*Z* = 4	0.2 × 0.03 × 0.01 mm

### Data collection

**Table:**

Bruker D8 Photon 100 area detector diffractometer	5655 independent reflections
Radiation source: microfocus sealed X-ray tube, Incoatec Iµs	4677 reflections with *I* > 2σ(*I*)
Mirror optics monochromator	*R*_int_ = 0.023
Detector resolution: 10.42 pixels mm^-1^	θ_max_ = 30.6°, θ_min_ = 2.5°
ω and φ shutterless scans	*h* = −10→10
Absorption correction: multi-scan (SADABS; Bruker, 2018)	*k* = −35→35
*T*_min_ = 0.554, *T*_max_ = 0.746	*l* = −14→15
37671 measured reflections	

### Refinement

**Table:**

Refinement on *F*^2^	Primary atom site location: structure-invariant direct methods
Least-squares matrix: full	Secondary atom site location: difference Fourier map
*R*[*F*^2^ > 2σ(*F*^2^)] = 0.018	Hydrogen site location: inferred from neighbouring sites
*wR*(*F*^2^) = 0.036	H-atom parameters constrained
*S* = 1.09	*w* = 1/[σ^2^(*F*_o_^2^) + (0.0071*P*)^2^ + 3.5107*P*] where *P* = (*F*_o_^2^ + 2*F*_c_^2^)/3
5655 reflections	(Δ/σ)_max_ = 0.001
264 parameters	Δρ_max_ = 0.92 e Å^−^^3^
0 restraints	Δρ_min_ = −1.09 e Å^−^^3^

### Special details

**Table:**

Geometry. All esds (except the esd in the dihedral angle between two l.s. planes) are estimated using the full covariance matrix. The cell esds are taken into account individually in the estimation of esds in distances, angles and torsion angles; correlations between esds in cell parameters are only used when they are defined by crystal symmetry. An approximate (isotropic) treatment of cell esds is used for estimating esds involving l.s. planes.

### Fractional atomic coordinates and isotropic or equivalent isotropic displacement parameters (Å^2^)

**Table:**

	*x*	*y*	*z*	*U*_iso_*/*U*_eq_	
Au1	0.75297 (2)	−0.00058 (2)	0.64453 (2)	0.01001 (2)	
O1	0.4872 (2)	0.22687 (7)	0.35894 (15)	0.0181 (3)	
O4	1.0437 (3)	−0.20014 (8)	0.49251 (17)	0.0272 (4)	
O3	0.9998 (2)	−0.20622 (7)	0.28226 (15)	0.0170 (3)	
O2	0.4569 (3)	0.20965 (8)	0.56671 (17)	0.0271 (4)	
N6	0.7546 (3)	0.04933 (9)	0.88472 (19)	0.0213 (4)	
N1	0.8122 (3)	−0.04540 (8)	0.48884 (17)	0.0141 (4)	
N5	0.6697 (3)	0.05028 (8)	0.78531 (18)	0.0180 (4)	
N4	0.7094 (4)	−0.07941 (11)	0.9575 (2)	0.0327 (5)	
N2	0.8539 (3)	−0.06048 (8)	0.76482 (18)	0.0173 (4)	
N7	0.8264 (4)	0.05242 (11)	0.9828 (2)	0.0346 (6)	
N3	0.7753 (3)	−0.06848 (9)	0.86238 (19)	0.0211 (4)	
C1	0.6763 (3)	0.05386 (8)	0.50946 (19)	0.0097 (4)	
C2	0.6095 (3)	0.10502 (9)	0.5321 (2)	0.0144 (4)	
H2	0.591225	0.116874	0.616458	0.017*	
C6	0.7021 (3)	0.03570 (9)	0.3866 (2)	0.0119 (4)	
C7	0.7724 (3)	−0.01977 (9)	0.3753 (2)	0.0120 (4)	
C10	0.9087 (3)	−0.12530 (9)	0.3809 (2)	0.0135 (4)	
C12	0.4984 (3)	0.19541 (9)	0.4620 (2)	0.0164 (4)	
C9	0.8673 (3)	−0.10048 (9)	0.2653 (2)	0.0156 (4)	
H9	0.885418	−0.119718	0.188635	0.019*	
C8	0.7995 (3)	−0.04760 (9)	0.2623 (2)	0.0152 (4)	
H8	0.771619	−0.030465	0.183738	0.018*	
C5	0.6615 (3)	0.07016 (9)	0.2849 (2)	0.0159 (4)	
H5	0.679171	0.058026	0.200723	0.019*	
C17	0.9570 (4)	−0.30317 (11)	0.3331 (3)	0.0324 (6)	
H17A	0.835596	−0.301253	0.287316	0.049*	
H17B	0.936999	−0.297241	0.423392	0.049*	
H17C	1.013575	−0.339051	0.320534	0.049*	
C4	0.5950 (3)	0.12224 (9)	0.3074 (2)	0.0161 (4)	
H4	0.567987	0.145929	0.238554	0.019*	
C3	0.5680 (3)	0.13978 (9)	0.4310 (2)	0.0139 (4)	
C16	1.0875 (3)	−0.26013 (9)	0.2839 (2)	0.0194 (5)	
H16A	1.203416	−0.259024	0.337608	0.023*	
H16B	1.124940	−0.269813	0.197057	0.023*	
C15	0.9899 (3)	−0.18128 (9)	0.3932 (2)	0.0156 (4)	
C13	0.4230 (4)	0.28268 (10)	0.3779 (2)	0.0220 (5)	
H13A	0.324410	0.283170	0.443070	0.026*	
H13B	0.366153	0.296654	0.298177	0.026*	
C11	0.8795 (3)	−0.09698 (9)	0.4924 (2)	0.0140 (4)	
H11	0.906730	−0.113793	0.571430	0.017*	
C14	0.5832 (4)	0.31882 (11)	0.4186 (3)	0.0338 (7)	
H14A	0.678520	0.319531	0.352664	0.051*	
H14B	0.640129	0.304889	0.497250	0.051*	
H14C	0.535687	0.355673	0.432548	0.051*	

### Atomic displacement parameters (Å^2^)

**Table:**

	*U*^11^	*U*^22^	*U*^33^	*U*^12^	*U*^13^	*U*^23^
Au1	0.01148 (3)	0.01142 (4)	0.00713 (3)	−0.00123 (3)	0.00055 (2)	−0.00031 (3)
O1	0.0228 (8)	0.0159 (8)	0.0156 (8)	0.0037 (6)	0.0019 (6)	0.0042 (6)
O4	0.0454 (12)	0.0202 (9)	0.0159 (8)	0.0049 (8)	−0.0039 (8)	−0.0024 (7)
O3	0.0211 (8)	0.0162 (8)	0.0137 (8)	0.0017 (6)	0.0006 (6)	−0.0042 (6)
O2	0.0426 (11)	0.0221 (9)	0.0171 (9)	0.0038 (8)	0.0093 (8)	0.0025 (7)
N6	0.0256 (11)	0.0225 (10)	0.0158 (9)	0.0013 (8)	0.0036 (8)	−0.0022 (8)
N1	0.0139 (8)	0.0166 (9)	0.0119 (8)	−0.0039 (7)	0.0014 (7)	−0.0030 (7)
N5	0.0201 (9)	0.0203 (9)	0.0138 (9)	0.0059 (8)	0.0011 (7)	−0.0039 (7)
N4	0.0417 (14)	0.0388 (14)	0.0178 (11)	−0.0078 (11)	0.0049 (10)	0.0061 (10)
N2	0.0197 (9)	0.0166 (9)	0.0155 (9)	0.0067 (7)	−0.0008 (7)	0.0008 (7)
N7	0.0392 (14)	0.0469 (15)	0.0175 (11)	0.0068 (12)	−0.0050 (10)	−0.0075 (10)
N3	0.0256 (10)	0.0210 (10)	0.0165 (10)	−0.0017 (8)	−0.0041 (8)	−0.0010 (8)
C1	0.0081 (8)	0.0102 (9)	0.0109 (9)	−0.0026 (7)	0.0006 (7)	0.0032 (7)
C2	0.0130 (9)	0.0158 (10)	0.0144 (10)	−0.0032 (8)	0.0007 (8)	0.0028 (8)
C6	0.0105 (9)	0.0132 (9)	0.0121 (9)	−0.0032 (7)	0.0002 (7)	0.0004 (7)
C7	0.0104 (9)	0.0162 (9)	0.0095 (9)	−0.0039 (7)	0.0004 (7)	−0.0005 (7)
C10	0.0131 (9)	0.0138 (9)	0.0136 (10)	−0.0046 (8)	0.0018 (7)	−0.0024 (8)
C12	0.0154 (10)	0.0174 (10)	0.0165 (10)	−0.0025 (8)	0.0022 (8)	0.0043 (8)
C9	0.0188 (10)	0.0164 (10)	0.0117 (9)	−0.0050 (8)	0.0019 (8)	−0.0048 (8)
C8	0.0176 (10)	0.0179 (10)	0.0100 (9)	−0.0046 (8)	0.0009 (8)	−0.0013 (8)
C5	0.0195 (10)	0.0173 (10)	0.0109 (9)	−0.0030 (8)	0.0002 (8)	0.0014 (8)
C17	0.0302 (14)	0.0163 (12)	0.0509 (18)	−0.0024 (10)	0.0096 (13)	−0.0062 (12)
C4	0.0165 (10)	0.0173 (10)	0.0146 (10)	−0.0035 (8)	0.0000 (8)	0.0044 (8)
C3	0.0119 (9)	0.0141 (9)	0.0157 (10)	−0.0026 (8)	0.0002 (7)	0.0021 (8)
C16	0.0200 (11)	0.0173 (11)	0.0209 (11)	0.0029 (9)	0.0033 (9)	−0.0052 (9)
C15	0.0175 (10)	0.0143 (10)	0.0150 (10)	−0.0036 (8)	0.0014 (8)	−0.0033 (8)
C13	0.0255 (12)	0.0182 (11)	0.0224 (12)	0.0100 (9)	0.0046 (10)	0.0049 (9)
C11	0.0127 (9)	0.0159 (10)	0.0135 (10)	−0.0039 (8)	0.0018 (7)	−0.0019 (8)
C14	0.0397 (16)	0.0146 (11)	0.0473 (18)	0.0045 (11)	0.0031 (14)	0.0010 (11)

### Geometric parameters (Å, º)

**Table:**

Au1—C1	2.027 (2)	C10—C9	1.394 (3)
Au1—N1	2.0335 (18)	C10—C15	1.498 (3)
Au1—N5	2.0418 (19)	C12—C3	1.494 (3)
Au1—N2	2.0674 (19)	C9—C8	1.388 (3)
O1—C12	1.339 (3)	C9—H9	0.9500
O1—C13	1.463 (3)	C8—H8	0.9500
O4—C15	1.204 (3)	C5—C4	1.388 (3)
O3—C15	1.328 (3)	C5—H5	0.9500
O3—C16	1.466 (3)	C17—C16	1.505 (4)
O2—C12	1.205 (3)	C17—H17A	0.9800
N6—N7	1.150 (3)	C17—H17B	0.9800
N6—N5	1.202 (3)	C17—H17C	0.9800
N1—C11	1.357 (3)	C4—C3	1.395 (3)
N1—C7	1.382 (3)	C4—H4	0.9500
N4—N3	1.150 (3)	C16—H16A	0.9900
N2—N3	1.198 (3)	C16—H16B	0.9900
C1—C2	1.369 (3)	C13—C14	1.499 (4)
C1—C6	1.390 (3)	C13—H13A	0.9900
C2—C3	1.397 (3)	C13—H13B	0.9900
C2—H2	0.9500	C11—H11	0.9500
C6—C5	1.397 (3)	C14—H14A	0.9800
C6—C7	1.459 (3)	C14—H14B	0.9800
C7—C8	1.395 (3)	C14—H14C	0.9800
C10—C11	1.390 (3)		
			
C1—Au1—N1	81.03 (8)	C4—C5—C6	119.7 (2)
C1—Au1—N5	91.82 (8)	C4—C5—H5	120.2
N1—Au1—N5	172.30 (8)	C6—C5—H5	120.2
C1—Au1—N2	172.51 (8)	C16—C17—H17A	109.5
N1—Au1—N2	92.15 (8)	C16—C17—H17B	109.5
N5—Au1—N2	95.13 (8)	H17A—C17—H17B	109.5
C12—O1—C13	116.48 (18)	C16—C17—H17C	109.5
C15—O3—C16	115.97 (18)	H17A—C17—H17C	109.5
N7—N6—N5	173.8 (3)	H17B—C17—H17C	109.5
C11—N1—C7	121.18 (19)	C5—C4—C3	120.0 (2)
C11—N1—Au1	124.28 (15)	C5—C4—H4	120.0
C7—N1—Au1	114.51 (15)	C3—C4—H4	120.0
N6—N5—Au1	118.70 (16)	C4—C3—C2	119.9 (2)
N3—N2—Au1	119.17 (16)	C4—C3—C12	122.8 (2)
N4—N3—N2	174.7 (3)	C2—C3—C12	117.3 (2)
C2—C1—C6	120.80 (19)	O3—C16—C17	112.3 (2)
C2—C1—Au1	125.05 (16)	O3—C16—H16A	109.1
C6—C1—Au1	114.12 (15)	C17—C16—H16A	109.1
C1—C2—C3	119.9 (2)	O3—C16—H16B	109.1
C1—C2—H2	120.1	C17—C16—H16B	109.1
C3—C2—H2	120.1	H16A—C16—H16B	107.9
C1—C6—C5	119.8 (2)	O4—C15—O3	124.9 (2)
C1—C6—C7	115.39 (19)	O4—C15—C10	123.1 (2)
C5—C6—C7	124.8 (2)	O3—C15—C10	112.00 (19)
N1—C7—C8	119.5 (2)	O1—C13—C14	111.2 (2)
N1—C7—C6	114.87 (18)	O1—C13—H13A	109.4
C8—C7—C6	125.7 (2)	C14—C13—H13A	109.4
C11—C10—C9	119.5 (2)	O1—C13—H13B	109.4
C11—C10—C15	116.8 (2)	C14—C13—H13B	109.4
C9—C10—C15	123.7 (2)	H13A—C13—H13B	108.0
O2—C12—O1	124.7 (2)	N1—C11—C10	120.3 (2)
O2—C12—C3	123.8 (2)	N1—C11—H11	119.9
O1—C12—C3	111.5 (2)	C10—C11—H11	119.9
C8—C9—C10	119.9 (2)	C13—C14—H14A	109.5
C8—C9—H9	120.1	C13—C14—H14B	109.5
C10—C9—H9	120.1	H14A—C14—H14B	109.5
C9—C8—C7	119.7 (2)	C13—C14—H14C	109.5
C9—C8—H8	120.2	H14A—C14—H14C	109.5
C7—C8—H8	120.2	H14B—C14—H14C	109.5
			
C6—C1—C2—C3	−0.6 (3)	C7—C6—C5—C4	180.0 (2)
Au1—C1—C2—C3	177.50 (15)	C6—C5—C4—C3	−0.4 (3)
C2—C1—C6—C5	0.7 (3)	C5—C4—C3—C2	0.5 (3)
Au1—C1—C6—C5	−177.58 (16)	C5—C4—C3—C12	179.5 (2)
C2—C1—C6—C7	−179.42 (19)	C1—C2—C3—C4	0.0 (3)
Au1—C1—C6—C7	2.3 (2)	C1—C2—C3—C12	−179.05 (19)
C11—N1—C7—C8	0.9 (3)	O2—C12—C3—C4	172.6 (2)
Au1—N1—C7—C8	−177.14 (16)	O1—C12—C3—C4	−7.0 (3)
C11—N1—C7—C6	−179.65 (18)	O2—C12—C3—C2	−8.4 (3)
Au1—N1—C7—C6	2.4 (2)	O1—C12—C3—C2	171.98 (19)
C1—C6—C7—N1	−3.1 (3)	C15—O3—C16—C17	76.9 (3)
C5—C6—C7—N1	176.8 (2)	C16—O3—C15—O4	−1.5 (3)
C1—C6—C7—C8	176.4 (2)	C16—O3—C15—C10	176.64 (18)
C5—C6—C7—C8	−3.8 (3)	C11—C10—C15—O4	−6.3 (3)
C13—O1—C12—O2	1.2 (3)	C9—C10—C15—O4	171.9 (2)
C13—O1—C12—C3	−179.18 (19)	C11—C10—C15—O3	175.56 (19)
C11—C10—C9—C8	0.8 (3)	C9—C10—C15—O3	−6.3 (3)
C15—C10—C9—C8	−177.4 (2)	C12—O1—C13—C14	84.2 (3)
C10—C9—C8—C7	−0.3 (3)	C7—N1—C11—C10	−0.4 (3)
N1—C7—C8—C9	−0.5 (3)	Au1—N1—C11—C10	177.39 (15)
C6—C7—C8—C9	−179.9 (2)	C9—C10—C11—N1	−0.4 (3)
C1—C6—C5—C4	−0.2 (3)	C15—C10—C11—N1	177.85 (19)
